# Experimental infection model for vibriosis in Dover sole (*Solea solea*) larvae as an aid in studying its pathogenesis and alternative treatments

**DOI:** 10.1186/s13567-018-0520-3

**Published:** 2018-02-26

**Authors:** Evelien De Swaef, Maaike Vercauteren, Luc Duchateau, Freddy Haesebrouck, Annemie Decostere

**Affiliations:** 1 Department of Morphology, Faculty of Veterinary Medicine, Ghent University, Salisburylaan 133, 9820 Merelbeke, Belgium; 2 Department of Pathology, Bacteriology and Avian Diseases, Faculty of Veterinary Medicine, Ghent University, Salisburylaan 133, 9820 Merelbeke, Belgium; 3 Biometrics Research Group, Faculty of Veterinary Medicine, Ghent University, Heidestraat 19, 9820 Merelbeke, Belgium

## Abstract

Severe economic losses due to diseases in marine larviculture may be linked to vibriosis. To better understand the pathogenesis of vibriosis and evaluate new ways to prevent and combat this important disease, there is a great need for reliable and reproducible experimental infection models. The present study aimed at developing a challenge model for vibriosis in Dover sole larvae and testing its applicability to study the effect of the probiotic treatment. For that purpose, larvae were challenged at 10 days post hatching with *Vibrio anguillarum* WT, *V. anguillarum* HI610 or *V. harveyi* WT. Following administration of *V. anguillarum* WT via immersion at 1 × 10^7^ colony forming units/mL, a larval mortality of 50% was observed at 17 days post-inoculation. In a next step, the probiotic potential of 371 isolates retrieved from Dover sole was assessed by screening for their inhibitory effects against *Vibrio* spp. and absence of haemolytic activity. One remaining isolate (*V. proteolyticus*) and *V. lentus*, known for its protective characteristics in seabass larvae, were further tested in vivo by means of the pinpointed experimental infection model. Neither isolate provided via the water or feed proved to be protective for the Dover sole larvae against challenge with *V. anguillarum* WT. This developed challenge model constitutes a firm basis to expedite basic and applied research regarding the pathogenesis and treatment of vibriosis as well as for studying the impact of (a)biotic components on larval health.

## Introduction

Dover sole (*Solea solea* L.) is greatly appreciated in high quality restaurants and has a high market value, making it a very promising candidate for European aquaculture [1, 2]. In addition, farmers developed a renewed interest in Dover sole aquaculture to diversify their operations due to indications of limited market growth for species such as sea bass (*Dicentrarchus labrax* L.) and sea bream (*Sparus aurata* L.) [1, 3]. Furthermore, a reliable sole production would reduce fishing pressure on wild Dover sole populations, whereby the main sole stocks only recently recovered after collapsing 20 years ago and are now at or close to being harvested sustainable [1, 3]. As for other marine teleost species, high larval mortality rates (especially during first feeding) and limited knowledge on the nutritional requirements result in juvenile scarcity for stocking purposes, being the main obstacle for large scale aquaculture [1, 4, 5].

One of the major causes for the low and unpredictable survival in marine larviculture are outbreaks of infectious diseases. Vibriosis is one of the most challenging bacterial diseases to tackle in these early life stages [6–8] and multiple publications stress the importance of pathogenic *Vibrio* species in hatcheries and their potential to cause disease [9–11]. The causative agents of vibriosis are bacteria belonging to the genus *Vibrio*, with *Vibrio anguillarum* being the most prominent member [7, 12]. Important contributions are made to prevent and control infectious diseases, in the past mainly focussing on the use of antimicrobial agents or chemical additives [13]. However, the emerging antimicrobial resistance, the potential transfer of antimicrobial resistance genes to fish or human pathogens [14] and the possibility that antimicrobials can enter the human food chain [15], stress the need to develop reliable alternatives. These latter should ensure a healthy microbial environment in the larval rearing tanks and hence decrease disease and mortality [16]. Various environmentally-friendly prophylactic disease treatments are currently being pinpointed for marine larvae including probiotics [17–19], prebiotics [20, 21] and immunostimulants [22], with hitherto no data are available on the use of such treatments including probiotics in Dover sole larvae. In addition, there is a clear paucity of information in our understanding of the mode of action of probiotics and their interaction with the aquatic organism especially in the marine larval stage [20, 23, 24]. To remediate this and to elucidate the mechanism by which these treatments exert their beneficial impact, more knowledge on how the bacterium interacts with its host and causes disease is needed. For that purpose, the availability of reliable experimental infection models is imperative. Only a limited number of studies succeeded in developing such models for marine fish larvae. Significant mortality was noted following challenge of turbot larvae (*Scophthalmus maximus* L.) with *V. anguillarum* HI610 [25] and sea bass larvae with *V. anguillarum* HI610 [26] or *V. harveyi* [27]. For Dover sole, an experimental multi well plate housing system was pinpointed [28]. However, a reproducible and reliable experimental infection model eliciting vibriosis is non-existing, hampering in-depth research on the interplay between *Vibrio* and its larval host.

In this respect, the present study aimed at developing the first experimental infection model for vibriosis in Dover sole larvae. In addition, the protective potential of probiotic candidates for Dover sole was evaluated in vitro and subsequently in vivo by means of the pinpointed challenge model.

## Materials and methods

All experiments were approved by the Ethical Committee of the Faculty of Veterinary Medicine and Bio-engineering Sciences, Ghent University (No. EC2015/28, EC2015/70 and EC2015/73).

### *Solea solea* larvae

*Solea solea* eggs were obtained from the Wageningen Marine Research (Ijmuiden, the Netherlands) and Stichting Zeeschelp (Kamperland, the Netherlands). Eggs were naturally spawned overnight and collected the next morning. The dead eggs were removed, whereafter transportation to the research facilities in natural seawater (32 g/L) occurred. Upon arrival, eggs were acclimatized with artificial seawater (ASW) of 34 g/L (Instant Ocean, Aquarium Systems, Mentor, Ohio) and further incubated herein under aeration. One day after arrival, dead eggs were removed and developing Dover sole eggs were disinfected with 1% H_2_O_2_ for 3 min [28]. After disinfection, eggs were kept in 400 mL aerated autoclaved artificial seawater (AASW, Instant Ocean) in glass bottles at 16 ± 1 °C, each bottle containing approximately 600 embryos. Housing the larvae was performed as described by [28]. Two days post-hatching (dph), larvae were placed individually in 24-well plates, incubated at 16 ± 1 °C and fed ad libitum with sterile *Artemia franciscana* nauplii (EG type; INVE Aquaculture NV, Belgium) every other day, starting from 6 dph onwards, except when indicated otherwise. Sterile *Artemia* cysts and nauplii were obtained through decapsulation [29]. Half of the well water was replaced every other day and all larvae were subjected to a circadian rhythm of 9 h light and 15 h darkness.

### Bacterial isolates

#### Experimental infection model

Three *Vibrio* strains were adopted. *Vibrio anguillarum* HI610 was originally isolated from diseased Atlantic cod (*Gadus morhua* L.) [30]. *Vibrio anguillarum* WT and *V. harveyi* WT strains were both procured from a disease outbreak in a French sea bass farm and subjected to minimal in vitro passaging.

#### In vitro selection of probiotic candidates

A total of 371 isolates retrieved from Dover sole larvae or the intestine of adults (both wild caught individuals and animals that were housed for 2–3 months in experimental facilities) were screened for their antagonism against *V. anguillarum* HI610, *V. anguillarum* WT or *V. harveyi* WT using the Kirby-Bauer disk diffusion method [31] as described in [32]. Briefly, the presence or absence of an inhibition zone surrounding disks immersed in the cultivated broth of the probiotic candidates following incubation, was recorded. The isolates eliciting growth inhibition were tested for their haemolytic activity by inoculating Marine Agar (MA, Scharlab S.L., Sentmenat, Spain) plates supplemented with 5% sheep blood (Oxoid Ltd, Hampshire, UK) with the cultivated broth of the probiotic candidates. Haemolytic activity was examined after 48 h incubation at 18 °C. Probiotic candidates exhibiting inhibition against at least one of the tested *Vibrio* strains and without haemolytic activity were identified by means of 16S rRNA gene sequencing. Therefore, the genomic DNA was extracted according to [33] and the 16S rRNA gene was amplified [34]. In short, amplification of the 16S rRNA gene was performed using the commercially available Qiagen Taq Mastermix and primers αβ-NOT (5′-TCAAACTAGGACCGAGTC-3′) and ωMB (5′-TACCTTGTTACTTCACCCCA-3′) as described by [35]. PCR products were sequenced using the BigDye Terminator sequencing kit (Applied Biosystems) and primers pD, Gamma*, 3 and O* [36]. Sequences were determined on an automatic DNA sequencer (ABI Prism 3100 Genetic analyser; Applied Biosystems) and identified using the program BLAST and the NCBI/GenBank. Species known to be potentially zoonotic were excluded from further experiments.

*Vibrio lentus* isolated from clinically healthy seabass larvae (10 dph) and proven to significantly reduce mortality of seabass larvae after challenge with *V. harveyi* WT [32], was also included as a probiotic candidate. *Vibrio lentus* showed in vitro inhibition against *V. anguillarum* HI610 and *V. harveyi* WT and proved to be non-haemolytic [32]. The inhibitory effect against *V. anguillarum* WT was tested as described above.

### In vivo experiments

For each experimental trial, Dover sole eggs from one single batch were used and a negative control group was included in which larvae underwent the same physical handling and water exchanges but without the addition of bacterial cells. Each group consisted of 96 larvae at 4 dph, divided over four 24-well plates filled with AASW. At the end of each experiment all remaining larvae were sacrificed by immersion in an overdose of MS 222 (tricaine methanesulfonate, Sigma-Aldrich, Diegem, Belgium).

#### Bacterial cultivation practices

All bacterial isolates were grown for 48 h at 18 °C on MA, followed by cultivation in tryptic soy broth (TSB, Becton, Dickinson and Company, New Jersey, USA) supplemented with 1.5% NaCl for 24 h at 18 °C. Cells were harvested by centrifugation at 3500 rpm for 10 min. The resulting pellet was washed twice with AASW and subsequently resuspended in AASW. Optical densities were determined using an ATB 1550 densitometer (BioMérieux, Marcy-l’Etoile, France). Bacterial titres were verified by making a tenfold dilution series in triplicate on MA plates, prior to administration.

#### Development of the experimental infection model

In the first challenge experiment, three groups were challenged with either *V. anguillarum* HI610, *V. anguillarum* WT or *V. harveyi* WT. The *Vibrio* strains were added to the well water of 10 dph larvae at a final concentration of 1 × 10^5^ colony forming units (CFU)/mL. In the second experiment, the same groups were included but the *Vibrio* strains were added so as to achieve a final concentration of 1 × 10^6^ CFU/mL. In the third experiment, only one group was challenged with *V. anguillarum* WT resulting in a final concentration of 1 × 10^7^ CFU/mL.

Six hours following the inoculation with the *Vibrio* strains, half of the well water was replaced. From the next day onwards, the normal feeding regime with sterile *Artemia* nauplii and water replacement every other day were started. Larval mortality was monitored daily up to 17 dph.

#### Assessment of the protective potential of probiotic candidates

##### Harmfulness of the probiotic candidates to Dover sole larvae

The harmfulness of the resulting probiotic candidate 1 and of *V. lentus* was tested in two separate experiments. Bacterial cells were added to the well water of larvae at 4, 6 and 8 dph resulting in a final concentration of 1 × 10^7^ CFU/mL. Larval mortality was monitored daily up to 17 dph. The standard body length of all remaining larvae was measured using an Olympus SZX7 stereomicroscope and cell D software (Soft imaging system, Olympus NV).

##### Protection of Dover sole larvae against challenge with *V. anguillarum* WT

In the first experiment, the larvae of two experimental groups were provided with probiotic candidate 1 or *V. lentus* via the well water on 4, 6 and 8 dph in a final concentration of 1 × 10^7^ CFU/mL. Subsequently, the larvae were challenged with *V. anguillarum* WT at a final concentration of 1 × 10^7^ CFU/mL at 10 dph. A third group (positive control) was inoculated with *V. anguillarum* WT without being previously administered a probiotic candidate.

In the second experiment the same experimental groups were included but probiotic candidates were supplied via the feed. Therefore, newly hatched sterile *Artemia* nauplii were incubated at 20 °C for 6 h in a suspension (1 × 10^7^ CFU/mL) of one of the two probiotic candidates. Subsequently, the *Artemia* nauplii were washed and fed to the Dover sole larvae at 5 and 7 dph. To evaluate the bacterial concentration present on the surface and inside of the *Artemia* nauplii, a subsample of at least 20 rinsed *Artemia* nauplii were homogenised and resuspended in 100 µL AASW. Bacterial titres were verified by making a tenfold dilution series of the homogenate on MA. From 9 dph onwards, the normal feeding regime with sterile *Artemia* nauplii every other day was started.

In both experiments, mortality was monitored daily up to 21 dph.

### Statistical analysis

For the experimental infection model, the survival (0–1) at the end of the study was compared between the three *Vibrio* strains (in different concentrations) and the negative control group using a logistic regression model. In the harmfulness study, the survival (0–1) of the negative control was compared with the probiotic candidates by a logistic regression model. The body length measurements of the larvae exposed to the probiotic candidates in comparison with the negative control group were analyzed within each experiment by a linear fixed effects model. To evaluate the protective potential of the probiotic candidates, administered via the water or the food, the survival (0–1) at the end of the study was compared between the negative control, the positive control and probiotic candidates by a logistic regression model.

All analyses were performed using SAS version 6.4. The global significance level of 5% was used but multiple comparisons significance levels were adjusted based on the Bonferroni correction method in order to compare the outcome of the challenges with the three *Vibrio* isolates with the negative control and to compare the probiotic candidate treatments, negative control and the positive control group (comparison wise significance level set at 0.05/3 = 0.0167).

## Results

### Experimental infection model

In the first challenge experiment to develop the infection model, no significant difference in survival at 17 dph was observed between the negative control group (survival of 91%) and the larvae inoculated with 10^5^ CFU/mL *V. anguillarum* HI610 (survival of 86%) (OR = 0.66, 95% CI [0.26;1.66], *p* = 0.367), *V. anguillarum* WT (survival of 84%) (OR = 0.56, 95% CI [0.23;1.37], *p* = 0.195) and *V. harveyi* (survival of 94%) (OR = 1.55, 95% CI [0.52;4.64], *p* = 0.423). In the second challenge experiment, no significant difference in survival at 17 dph was noted between the negative control group (survival of 90%) and the larvae inoculated with 10^6^ CFU/mL *V. anguillarum* HI610 (survival of 93%) (OR = 1.48, 95% CI [0.53;4.16], *p* = 0.448) or *V. harveyi* WT (survival of 86%) (OR = 0.90, 95% CI [0.36;2.27], *p* = 0.817). Following challenge with *V. anguillarum* WT at 10^6^ CFU/mL, a statistically significant reduction in survival to 61% (OR = 0.19, 95% CI [0.08;0.41], *p* < 0.001) was discerned. In the third experiment, a significantly reduced survival of 50% (*p* < 0.001) was detected between the larvae inoculated with *V. anguillarum* WT at 10^7^ CFU/mL and the negative control group (survival of 92%) [OR = 0.09, 95% CI [0.04;0.21], *p* < 0.001).

### Assessment of the protective potential of probiotic candidates

#### In vitro selection of probiotic candidates

None of the isolates retrieved from adult Dover sole displayed in vitro inhibition against one of the tested *Vibrio* strains. Four probiotic candidates recovered from Dover sole larvae were selected based on the presence of in vitro inhibition against the three tested *Vibrio* strains and the absence of haemolytic activity. Following 16S rRNA sequencing, three probiotic candidates were identified as *V. parahaemolyticus* with 99% sequence homology and hence excluded from further assays due to their potential zoonotic character. The remaining probiotic candidate was identified as *V. proteolyticus* with 99% sequence homology and further analysed in vivo for its protective potential.

*Vibrio lentus* isolated from seabass larvae demonstrated in vitro inhibition against *V. anguillarum* WT.

#### Harmfulness of the probiotic candidates to Dover sole larvae

In the first experiment, no significant difference in survival at 17 dph was observed between the negative control group (survival of 76%) and the larvae inoculated with *V. proteolyticus* (survival of 74%) (OR = 0.89 [95% CI 0.46;1.74], *p* = 0.739). Also in the second experiment, no significant difference in survival at 17 dph was observed between the negative control group (survival of 89%) and the larvae inoculated with *V. lentus* (survival of 88%) (OR = 0.89 [95% CI 0.46;1.74], *p* = 0.824). In both experiments the estimated odds ratios were close to one. No significant difference in standard body length was observed at 17 dph between the control group and the group inoculated with *V. proteolyticus* (*p* = 0.3223). By 17 dph, the larvae inoculated with *V. lentus* had a mean standard body length of 5570.49 µm (SD = 749.13 µm), which is significantly higher than what was noted in the larvae of the control group, 5000.06 µm (SD = 421.73 µm) (*p* = 0.006).

#### Protection of Dover sole larvae against challenge with *V. anguillarum* WT

In the first experiment, no significant differences in survival at 21 dph were observed between the positive control group (survival of 35%) and the larvae inoculated before challenge via the water with *V. proteolyticus* (survival of 37%) (OR = 1.05 [95% CI 0.57;1.91], *p* = 0.880) or with *V. lentus* (survival of 34%) (OR = 0.96, 95% CI [0.52;1.75], *p* = 0.880). A significant difference in survival at 21 dph was discerned between the negative control group (survival of 80%) and the larvae inoculated with *V. proteolyticus* (OR = 0.14 [95% CI 0.07;0.26], *p* < 0.001) or with *V. lentus* (OR = 0.14 [95% CI 0.07;0.26], *p* < 0.001) prior to challenge.

In the second experiment, no significant differences in survival at 21 dph were noted between the positive control group (survival of 52%) and the larvae that were inoculated via the feed before being challenged with *V. proteolyticus* (survival of 45%) (OR = 0.75 [95% CI 0.42;1.33], *p* = 0.312) or with *V. lentus* (survival of 49%) (OR = 0.88, 95% CI [0.50;1.57], *p* = 665). A significant difference in survival at 21 dph was noticed between the negative control group (survival of 72%) and the larvae inoculated with *V. proteolyticus* (OR = 0.32 [95% CI 0.17;0.59], *p* < 0.001) or with *V. lentus* (OR = 0.38 [95% CI 0.20;0.69], *p* = 0.001) before challenge. The bacterial concentration of the *Artemia* nauplii ranged between 1.5 × 10^7^ and 5 × 10^7^ CFU/mL for *V. proteolyticus* and between 2.5 × 10^7^ and 2 × 10^8^ CFU/mL for *V. lentus*.

## Discussion

Infectious diseases (e.g. vibriosis) are a major cause of marine larval mortality and various environmentally-friendly prophylactic treatments are currently being pinpointed including the use of pro- and prebiotics. However, very limited data on these alternative treatments are available for Dover sole. In the present study, an experimental challenge model using *V. anguillarum* WT was developed to reproduce vibriosis in Dover sole and therefore provide a building block to move forward in the assessment of novel therapies and the elucidation of the pathogenesis of vibriosis. This model was then used for evaluating the protective potential of two probiotic candidates, selected using in vitro criteria.

Only a limited number of studies focus on the development of challenge models in marine fish larvae. These may be performed by bioencapsulation of the pathogen in life feed [25, 37, 38] or challenge via the water. When pathogen delivery is performed via the water, the challenge experiments are mostly conducted in multiwell plate systems [27, 39–41]. In a minority of studies, larvae are housed in small groups in vials [26, 42]. However, in these vials an increase virulence of *V. anguillarum* depending on the number of dead larvae was observed [42]. This indicates that (remnants of) death larvae can have an impact on living animals or the pathogen, increasing variability between replicates. The challenge model as proposed in the current study draws on a multi well housing system that was developed previously [28]. Housing the larvae individually offers the advantage that the possible death of one larva has no effect on the other larvae, rendering these experiments more reproducible. Furthermore, the health status and behaviour of individually housed larvae may be monitored more easily. In the present study, the pathogenicity of three bacterial strains was evaluated and the strain and concentration eliciting around 50% mortality within 6 days following challenge selected. Indeed, the induced mortality needs to be sufficiently high to enable the investigation of the protective effect of prophylactic or curative treatments. On the other hand, a too severe challenge model is not appropriate neither as this would hinder detection of a possible protective capacity.

Vibriosis is reported as a cause of disease/mortality in as many as at least 48 species of marine fish [43]. The importance of pathogenic *Vibrio* species in hatcheries and their potential to cause disease is stressed by various authors [9–11]. *Vibrio anguillarum* is described as the causative agent of vibriosis in the young life stages of at least 12 fish species. The number of disease case reports in *S. solea* culture is limited and involves adults [44] and juveniles [45]. This scarce information on Dover sole health management is largely rooted in the fact that Dover sole aquaculture was initiated only relatively recently, underscoring the need for research on health and disease in this alternative aquaculture species. A *V. anguillarum* isolate not originally retrieved from sole was included that induced larval mortality in other marine species. Indeed, although fish species-specific virulence was described for different *Vibrio* species, several strains originating from a disease outbreak in (larvae of) one fish species were able to cause mortality in another [41, 46, 47]. To exemplify this, although *V. anguillarum* strain 87-9-117 was originally isolated from rainbow trout (*Oncorhynchus mykiss* Walbaum), it caused high mortality in sea bass larvae, indicating that there is no stringent host-specificity for vibriosis [47]. In the present study, normal survival was obtained for Dover sole larvae subsequent to inoculation with *V. harveyi* WT retrieved from diseased sea bass and pathogenic to sea bass larvae [27]. Indeed, in a previous study challenge with *V. harveyi* resulted in 70% mortality in sea bass larvae following administration at 1 × 10^5^ CFU/mL [32]. The latter is also a well-known causative agent of vibriosis in adults of the closely related Senegalese sole (*Solea senegalensis* Kaup) [48, 49]. Secondly, no increased mortality was observed following challenge of the Dover sole larvae with *V. anguillarum* HI610 at concentrations up to 1 × 10^6^ CFU/mL. This bacterial strain originating from diseased Atlantic cod larvae [30], elicited high mortality rates in several experimental challenge tests including yolk sac larvae of turbot [41, 47], halibut (*Hippoglossus hippoglossus* L.) [41, 47], Atlantic cod [41, 49] and seabass [26, 27]. Thirdly, challenge with *V. anguillarum* WT resulted in significant mortality in Dover sole larvae with an increased death rate noted following inoculation with a higher concentration [39% (1 × 10^6^ CFU/mL) vs 50% (1 × 10^7^ CFU/mL)]. Although retrieved from a different fish species that is sea bass, adequate virulence hence was established. These results again underscore the complexity of fish species-specific virulence of *V. anguillarum*, impeding extrapolation of experimental challenge models across species and warranting further research.

Although efforts were and are still being made to understand the physiological changes during stress events [50], research concerning welfare and pain awareness parameters in fish larvae remains practically nonexistent. Studies on predictive behavioural traits indicating severe suffering or imminent mortality are imperative and should allow to delineate humane endpoints for fish larvae. The individual housing of the larvae as is the case in the current experimental set-up, may be regarded as an aid in this research journey. In order to reduce the number of experimental animals used, a subsequent experiment with an increasing bacterial concentration was only performed when the lower bacterial titer did not generate sufficient mortality. In the third experiment, *V. anguillarum* WT was administered at a higher dose (1 × 10^7^ CFU/mL) since only this strain showed pathogenic potential when administered at a lower concentration (1 × 10^6^ CFU/mL). As no increased larval mortality was observed after inoculation with *V. anguillarum* HI610 and *V. harveyi* WT at 1 × 10^6^ CFU/mL, both isolates were omitted in the third experiment.

Once the standardized challenge model was developed, the protective potential of two probiotic candidates was evaluated. Probiotics are usually defined as products which contain viable non-pathogenic micro-organisms able to confer health benefits to the host [3]. The effectiveness of probiotic in terms of protection against bacterial pathogens especially was tested in juveniles and adult fish (reviewed in [18, 19]) but also a small number of studies on marine fish larvae were performed. The protective potential of various probiotic candidates involving turbot larvae was evaluated in bottles [51] or tanks [52, 53]. To our knowledge, only one study tested the protective potential of probiotic candidates in a challenge experiment using multi well plates (based on sea bass larvae, [32]), hereby highlighting the significance of the present study on Dover sole larvae.

Different probiotics were tested for flatfish species, mainly focusing on turbot, with only a limited number of studies involving fish larvae (turbot [25, 54–56], California halibut (*Paralichthys californicus* Ayres) [57], Dover sole [58], Senegalese sole [5, 59]). Considering sole species, Senegalese sole is widely studied and one probiotic *Shewanella putrefaciens* was put forward. Beneficial effects on growth, stress levels, onset of metamorphosis, intestinal flora and resistance against infection with *Photobacterium damselae* were found (reviewed in [60]). For Dover sole, only one probiotic was described (*Enterococcus faecium*, [58]), modulating amongst others growth and cortisol levels [61]. However, the protective potential of this probiotic against challenge with a pathogenic agent was not evaluated. In this study *V. lentus* and *V. proteolyticus* were evaluated as probiotic candidates. *Vibrio lentus* was described as the causative agent of skin lesions and mortality in wild octopus (*Octopus vulgaris* Cuvier) [62] but no pathogenic properties were observed in fish, following both intraperitoneal injection [62] or challenge via *Artemia* nauplii [63]. Also in the present study no decreased survival of the larvae was observed, but due to the large width of the 95% CI, a negative impact on fish larval health may not be fully excluded. However, at 17 dph a significant increase in standard body length of the larvae inoculated with *V. lentus* compared with the control group was noted. A beneficial growth effect has been correlated with the administration of probiotics via bioencapsulation to the life feed in fish larvae and juveniles [52, 64] but no such effect has been described in marine larvae after probiotic treatment via the water. Increased growth rates due to probiotic administration were linked to the production of beneficial dietary compounds or digestive enzymes [24]. The probiotic potential of *V. lentus* was previously tested in gnotobiotic sea bass larvae against *V. harveyi* WT, showing significantly decreased mortality rates [32]. In the present study, however, no such properties were seen in Dover sole larvae after challenge with *V. anguillarum* WT. No length measurements were performed by [32]. *Vibrio proteolyticus* can induce mortality in *Artemia* cultures [19] but no such characteristics have been reported in fish. Also in this study, no increased mortality nor impaired larval growth was observed after administration of this isolate. Beneficial effects of diet supplementation with *V. proteolyticus* on protein degradation were noted [65]. However, the potential beneficial role of *V. proteolyticus* as a probiotic agent has not yet been described in aquatic organisms. This renders the present study the first in its kind although no positive effects, observed as an increased survival after challenge with the pathogen or increased standard body length, were discerned.

To test the protective effect of the probiotic candidates during *V. anguillarum* WT infection, two routes of delivery, via the well water and via attachment to live food (*Artemia* nauplii), were tested. Adding the probiotic candidate through the rearing water maximizes the exposure of the larvae before the start of exogenous feeding and during the first days, when the food intake is limited [24, 66]. Furthermore, bath challenge may maximize the competitive advantage of added probiotics, as bacteria colonizing the intestines before first feeding may be able to persist amongst the autochthonous microflora [64, 67]. Exogenous feeding was started at 6 dph in Dover sole larvae and also the delivery of the probiotics via *Artemia* nauplii was studied. Delivery through bioencapsulation may be preferred in hatcheries where water exchange rates are high [24]. For bioencapsulation, lower amounts of probiotic components are needed compared to when these are added to the water, rendering this practice more feasible and economically more interesting. One may presume that delivery through the feed results in the probiotic residing longer in the intestine and hence may increase the probiotic protective potential. In addition, it was described that colonization of the gut increases during exogenous feeding, hereby resembling the microflora of the livefood [68]. Most probiotic studies focus only on one route of delivery, via life feed (e.g. [51]) or via the water (e.g. [5]), underscoring the completeness of this study.

In addition to its possible value for many other applications, this experimental infection model for vibriosis constitutes a firm basis to evaluate the impact of (a)biotic components on larval health. The model is also to be regarded as a powerful tool for investigating the pathogenesis of *V. anguillarum* infections in Dover sole larvae, evaluating curative or preventive treatments and elucidating their mode(s) of action. In this study, probiotic candidates were selected in vitro and assessed for their protective potential against *V. anguillarum* challenge in vivo but also prebiotic treatments may be evaluated by means of this model. Indeed, although immunostimulating properties are allocated to prebiotics [21], limited research on the potential protective effect of prebiotics against challenge with a known pathogen has been performed in marine fish larvae [69]. Next to aquaculture related research, this model also renders biological research concerning the impact of environmental components (e.g. microplastics or algal toxins released during harmful algal blooms) on the health of Dover sole larvae possible by evaluating their susceptibility to disease agents.

## Abbreviations

AASW: autoclaved artificial seawater
ASW: artificial seawater
CFU: colony forming units
dph: days post-hatching
MA: marine agar
TSB: tryptic soy broth
